# Supplementary material for: Proteomic Analysis to Understand the Promotive Effect of Ethanol on Soybean Growth Under Salt Stress
Source: Biology (Basel). 2024 Oct 24;13(11):861. doi: 10.3390/biology13110861 (PMC11591660; doi:10.3390/biology13110861)
Supplement: Supplementary file 1 [file biology-13-00861-s001.zip › Supplemental Figures.pdf]

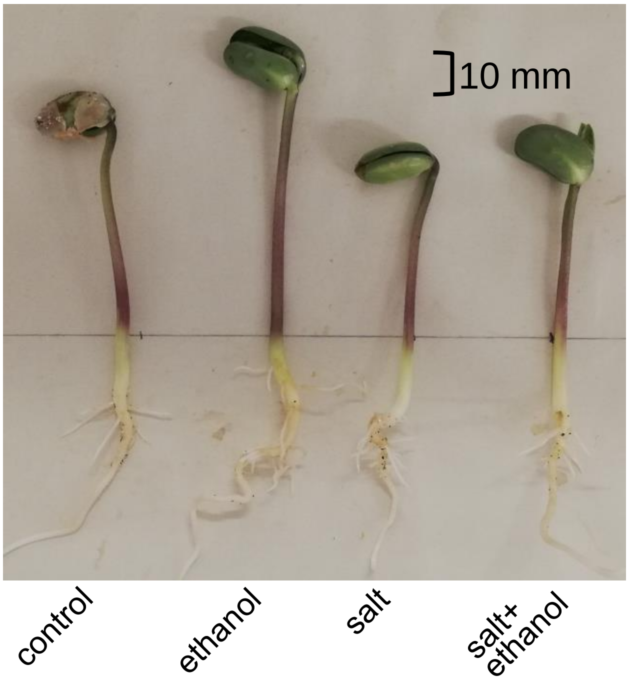

Figure S1. Photograph of soybean treated with and without ethanol as well as with and without salt stress. Soybean seeds were sown and 3-day-old seedlings were treated with or without 300 mM ethanol as well as with or without 150 mM NaCl for 2 days. The bar in the picture indicates 10 mm.

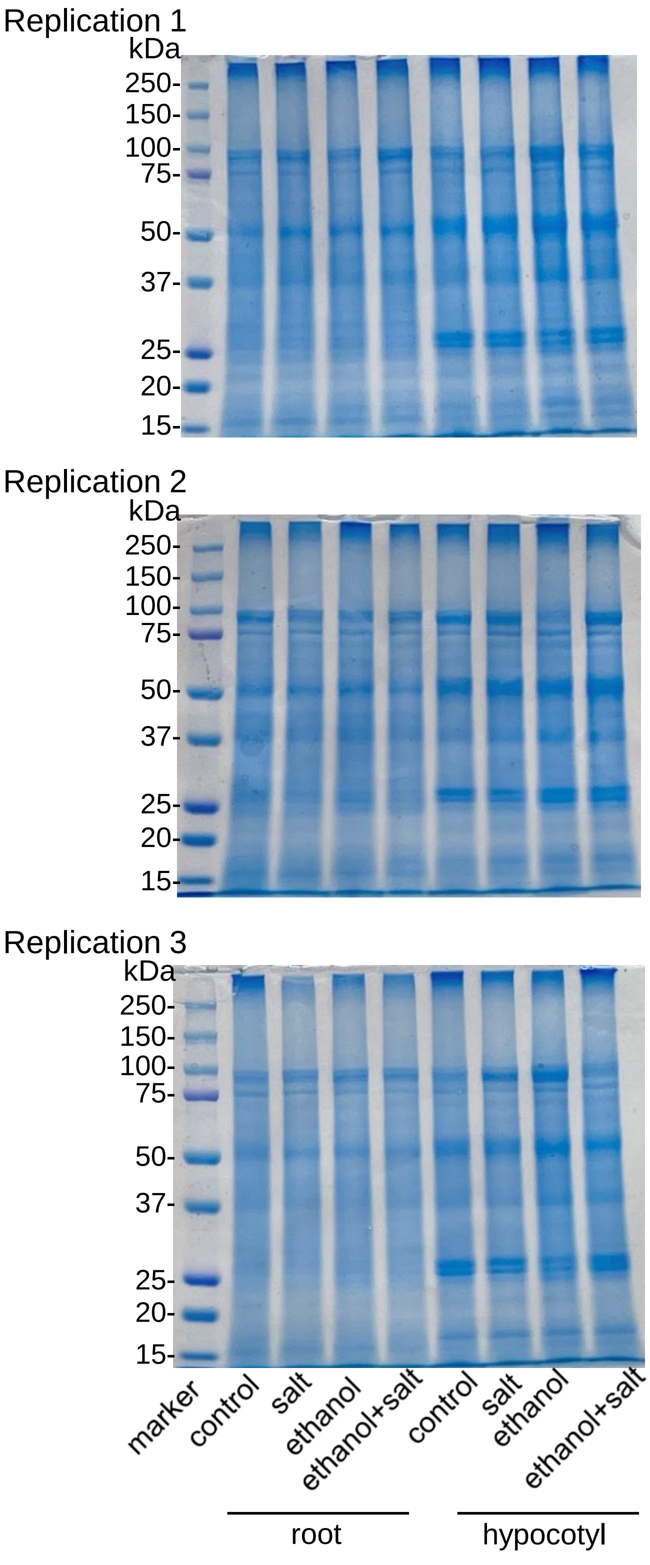

Figure S2. The gel pattern of proteins stained with the Coomassie-brilliant blue used for immunoblot analysis. Experiments were performed with 3 biological replicates for each treatment. Quantified proteins (10 µg) from root and hypocotyl were separated by electrophoresis on a 10% SDS-polyacrylamide gel. Coomassie-brilliant blue staining was used as a loading control.

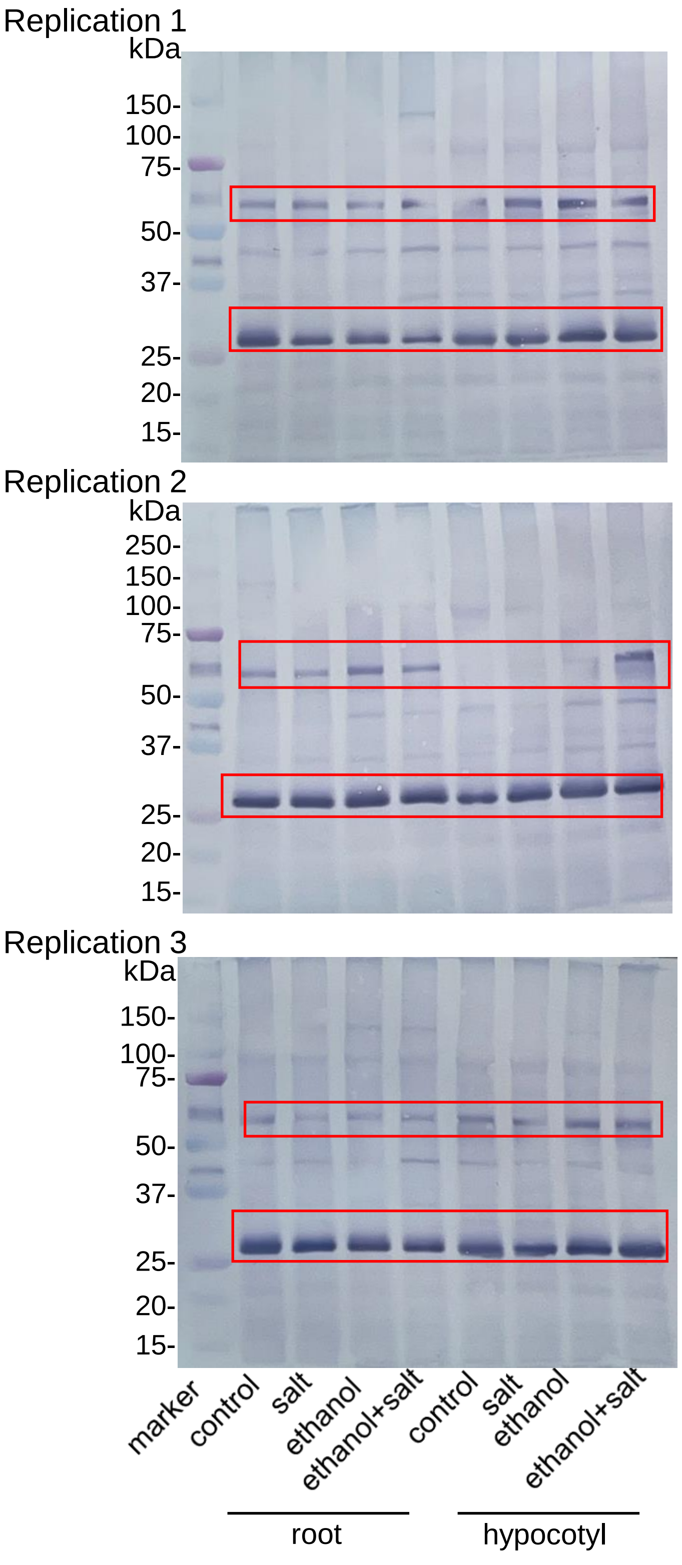

Figure S3. Immunoblot patterns of the entire PVDF membranes reacted with anti-ascorbate peroxidase antibody, which is used in Figure 5A and B.

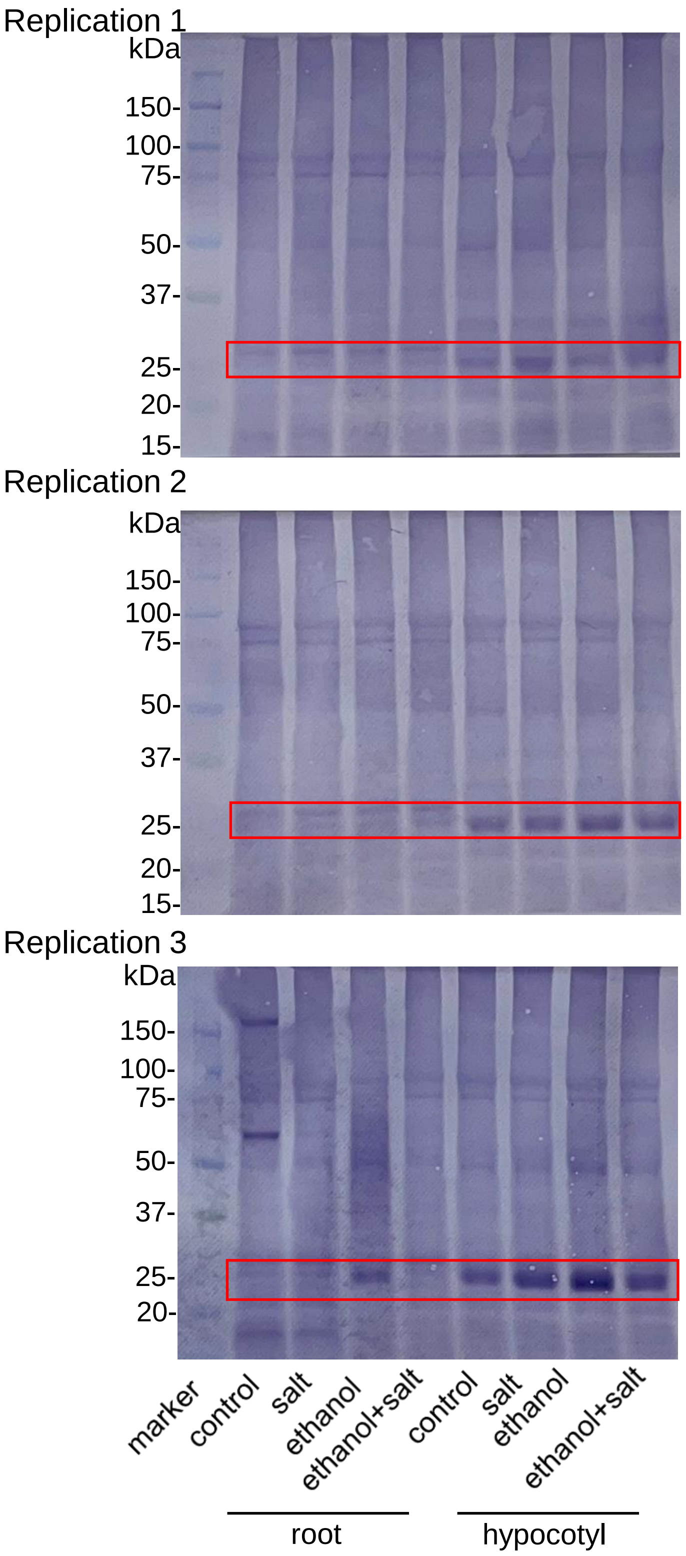

Figure S4. Immunoblot patterns of the entire PVDF membranes reacted with anti-peroxiredoxin antibody, which is used in Figure 5C.

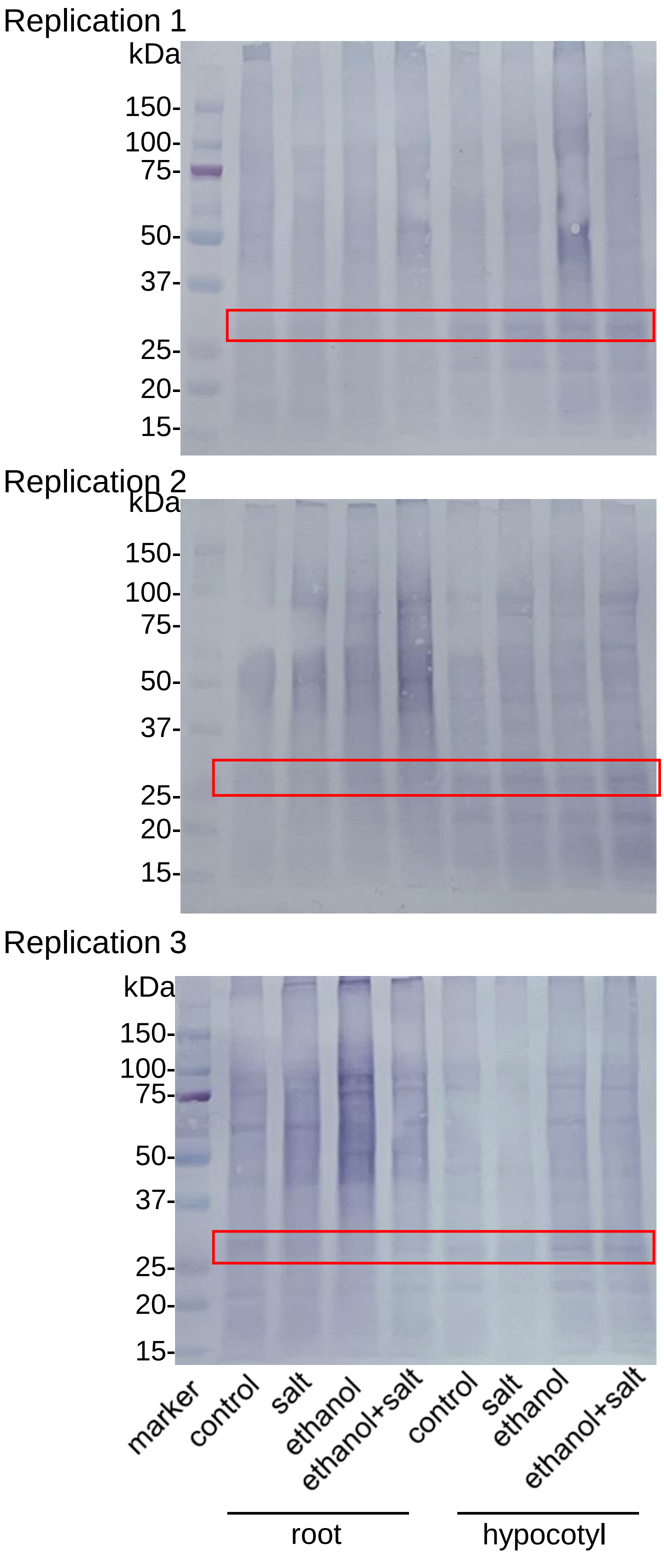

Figure S5. Immunoblot patterns of the entire PVDF membranes reacted with anti-xyloglucan xyloglucosyl transferase antibody, which is used in Figure 6A.

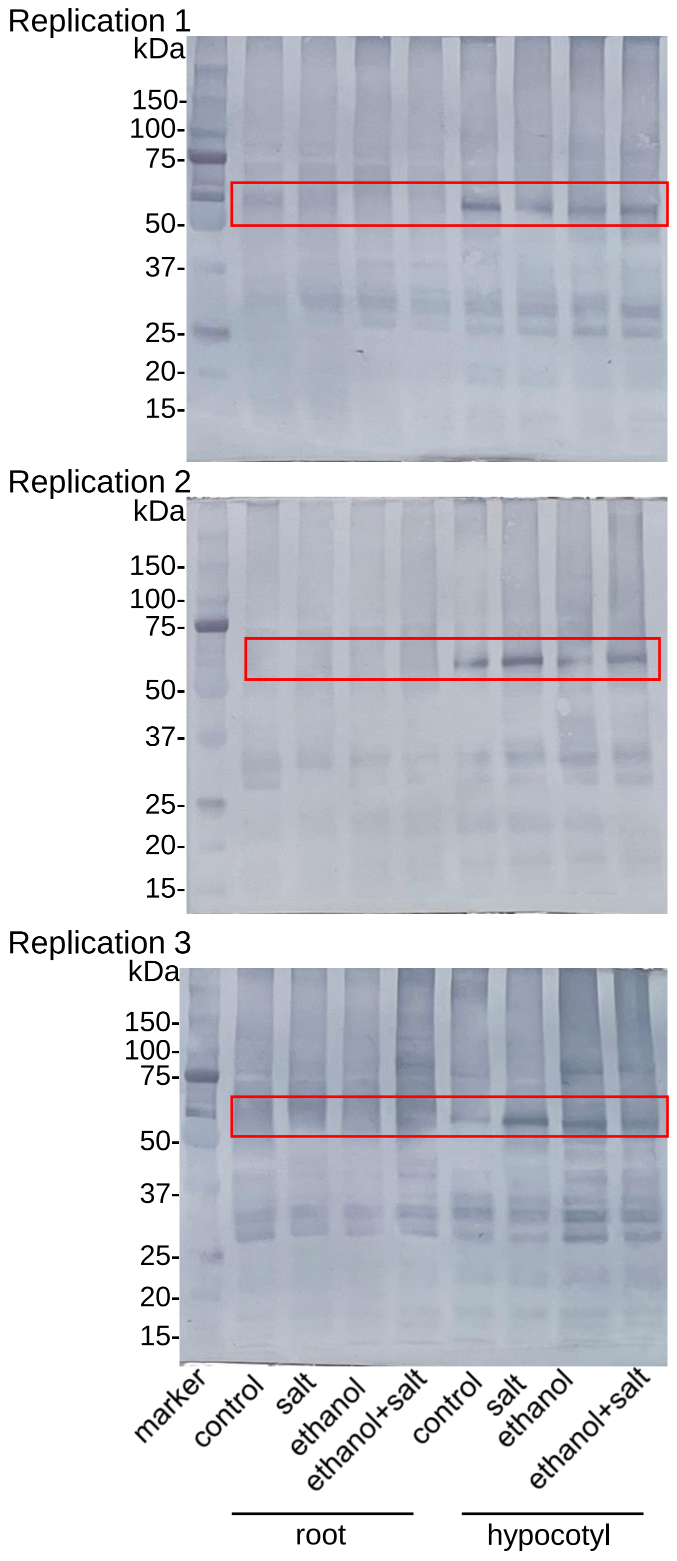

Figure S6. Immunoblot patterns of the entire PVDF membranes reacted with anti-cellulose synthase antibody, which is used in Figure 6B.

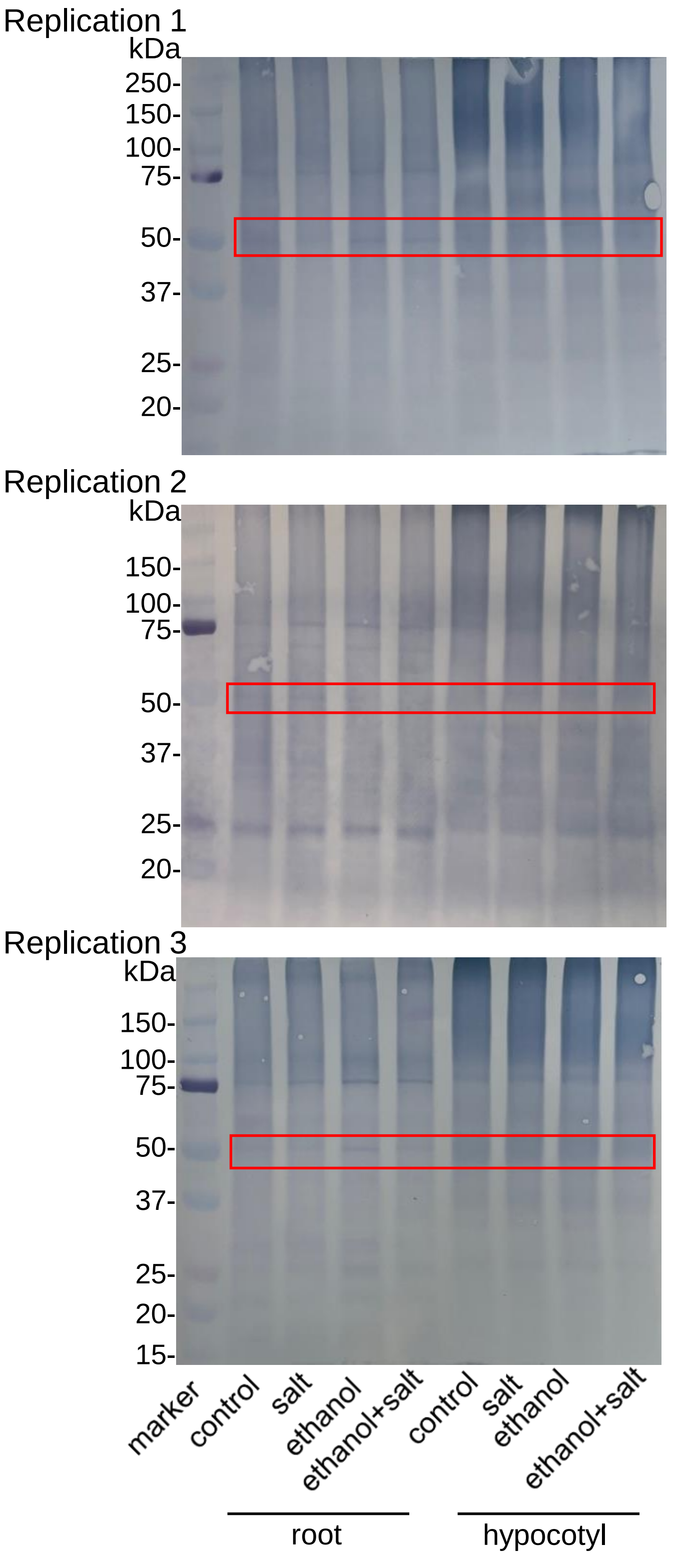

Figure S7. Immunoblot patterns of the entire PVDF membranes reacted with anti-H^+-ATPase antibody, which is used in Figure 6C.
